# A Novel Nonsense *CDK5RAP2* Mutation in a Somali Child With Primary Microcephaly and Sensorineural Hearing Loss

**DOI:** 10.1002/ajmg.a.35558

**Published:** 2012-08-10

**Authors:** Alistair T Pagnamenta, Jennie E Murray, Grace Yoon, Elham Sadighi Akha, Victoria Harrison, Louise S Bicknell, Kaseem Ajilogba, Helen Stewart, Usha Kini, Jenny C Taylor, David A Keays, Andrew P Jackson, Samantha JL Knight

**Affiliations:** 1 NIHR Biomedical Research Centre, Oxford and Wellcome Trust Centre for Human Genetics, University of OxfordOxford, UK; 2 MRC Human Genetics Unit at the MRC Institute of Genetics & Molecular Medicine, University of Edinburgh, Western General HospitalEdinburgh, UK; 3 Division of Clinical and Metabolic Genetics; Division of Neurology, The Hospital for Sick Children, University of TorontoToronto, Ontario, Canada; 4 Department of Clinical Genetics, Oxford University Hospitals NHS TrustOxford, UK; 5 Department of Paediatric Radiology, Royal Hospital for Sick Children, NHS LothianEdinburgh, UK; 6 Research Institute of Molecular PathologyVienna, Austria

**Keywords:** *CDK5RAP2*, MCPH3, microcephaly, deafness, SNP array

## Abstract

Primary microcephaly is a genetically heterogeneous condition characterized by reduced head circumference (−3 SDS or more) and mild-to-moderate learning disability. Here, we describe clinical and molecular investigations of a microcephalic child with sensorineural hearing loss. Although consanguinity was unreported initially, detection of 13.7 Mb of copy neutral loss of heterozygosity (cnLOH) on chromosome 9 implicated the *CDK5RAP2* gene. Targeted sequencing identified a homozygous E234X mutation, only the third mutation to be described in *CDK5RAP2*, the first in an individual of non-Pakistani descent. Sensorineural hearing loss is not generally considered to be consistent with autosomal recessive microcephaly and therefore it seems likely that the deafness in this individual is caused by the co-occurrence of a further gene mutation, independent of *CDK5RAP2*. Nevertheless, further detailed clinical descriptions of rare *CDK5RAP2* patients, including hearing assessments will be needed to resolve fully the phenotypic range associated with mutations in this gene. This study also highlights the utility of SNP-array testing to guide disease gene identification where an autosomal recessive condition is plausible. © 2012 Wiley Periodicals, Inc.

## INTRODUCTION

Mutations in *ASPM* and *WDR62* account for the majority of patients with primary microcephaly [Bond et al., 2002; Nicholas et al., 2010]. Five other causative genes are known but have only been reported in a few individuals (for recent review see [Mahmood et al., 2011]). A recent study suggests *CEP135* may be responsible for an eighth microcephaly locus on chromosome 4q12 [Hussain et al., 2012]. The fact that the majority of these microcephaly genes were mapped using consanguineous kindreds from Pakistan highlights the importance of the autozygosity mapping strategy in the understanding of this rare condition. However, as a consequence, it is unclear what clinical relevance these genes have in other populations.

*CDK5RAP2* is responsible for one of the rarest forms of primary microcephaly (MCPH3), with only two different mutations published to date, in three independent families originating from northern Pakistan [Bond et al., 2005; Hassan et al., 2007] (Table I). A recent study of primary microcephaly patients from 112 consanguineous Iranian pedigrees did not show any linkage to this locus, indicating that *CDK5RAP2* mutations may be rare even in consanguineous families [Darvish et al., 2010]. In common with other microcephaly genes, *CDK5RAP2* appears to be involved with centrosomal function: an inversion mutation in mice leads to abnormal spindle poles, spontaneous aneuploidy and neurogenic defects, resulting in microcephaly in some strains [Lizarraga et al., 2010]. There is also evidence that *CDK5RAP2* has undergone positive selection and may have been a genetic factor leading to the evolutionary increase in human brain size [Montgomery et al., 2011].

**TABLE I:** Comparison of Published Patients With *CDK5RAP2* Mutations

Refs.	Mutation based on NM_018249.4 (predicted effect on protein)	Ethnicity/level of parental consanguinity	Patient ID/gender	Degree of microcephaly	Learning disability	Miscellaneous
Described here	c.700G>T (p.E234X)	Somali/second cousin	AJ213/female	OFC: 30.0 cm at birth (−3.7 SDS), 36.5 cm at 10 months (−8.0 SDS), 38.5 cm at 16 months (−7.7 SDS), 41.5 cm at 6 years (−8.9 SDS)	Mild	Sensorineural hearing loss. Passed hearing test at birth so postnatal onset. No family history reported. Significant reflux during the first 4 months of life leading to gastrostomy, later advanced to gastro-jejunostomy
Bond et al. [2005 ] (Clinical details for Pedigree 1 given by Moynihan et al. [2000])	Pedigree 1 has c.246T>A (p.Y82X)	Northern Pakistan/first cousin^a^	VI-2/male	Microcephaly present at birth; 6–8 SDS below age- and sex-related means	Mild	—
			VI-3/female		Moderate	Profound congenital sensorineural deafness and infrequent tonic/clonic fits
			VI-7/female		Mild (WISC-R full-scale IQ of 86^b^)	—
			VI-8/female		Mild (WISC-R full-scale IQ of 89^b^)	Developed acute lymphoblastic leukemia
	Pedigree 2 has c.4005-15A>G (novel splice acceptor—addition of four new amino acids and then a termination codon)	Northern Pakistan/first cousin^a^	Two female cousins (no IDs given)	Both had congenital microcephaly with late closing fontanels. One patient was −7 SDS below age- and sex-related means at 11y. The other was −5 SDS below age- and sex-related means at 4y^c^	Moderate^c^	Low birth weight: 1.9 kg at term. Subsequent growth normal. No deafness, fits or spasticity in either individual^c^
Hassan et al. [2007]	c.246T>A (p.Y82X)	Northern Pakistan (Kashmir)/first cousins^a^	V-2/female	Microcephaly present at birth; 4–7 SDS below age- and sex-related means	Mild to moderate, with IQs all in the range of 51–65	—
			V-3/female			
			V-4/male			
			V-5/male			

WISC-R, Weschsler Intelligence Scale for Children-Revised; SDS, standard deviations; OFC, occipito-frontal head circumference. For the patient described here, it appears that the microcephaly shows some degree of progression; the other published patients do not present OFC measurements at multiple time points and so it is not possible to determine whether this is a common feature of *CDK5RAP2* patients.

a Additional consanguineous loops present in the pedigree.

b IQs reported by Heney et al. [1992].

c Personal communication, Professor C.G. Woods.

Here, we describe an individual of Somali descent with primary microcephaly and sensorineural hearing loss. Although the family was initially reported to be non-consanguineous, detection of two regions of copy neutral loss of heterozygosity (cnLOH) led to further molecular investigation of the *CDK5RAP2* gene.

## CLINICAL REPORT

**FIG. 1:**
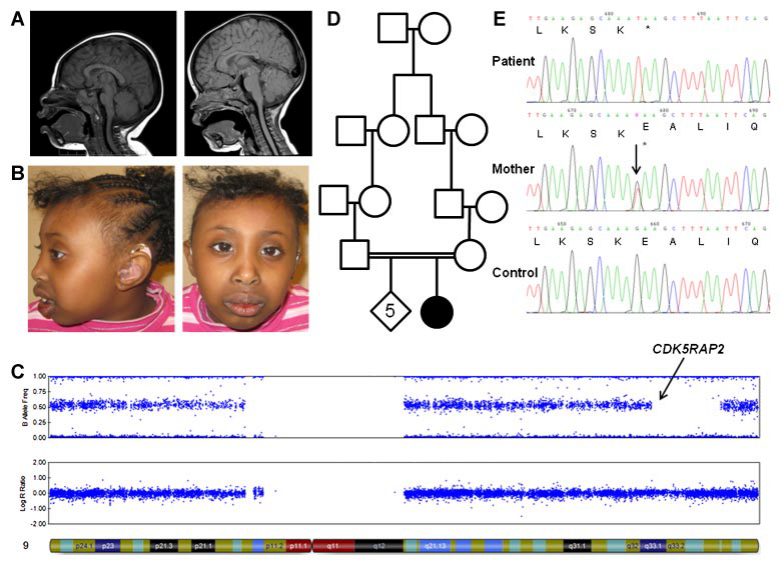
A: Sagittal T1SE MRI images of patient at 15 months (on left) compared to a normal age- and gender-matched child (on right), demonstrating cranio-facial disproportion characteristic of microcephaly. B: Photographs taken at age 6 years, shown with parental consent, indicating microcephaly and sloping forehead. C: SNP-array data for chromosome 9 showing a 13.7 Mb region of copy-neutral loss of heterozygosity at chr9:120,050,463–133,809,775 (GRCh19/hg37). In combination with a second region of cnLOH (chr7:9,870,471–27,658,801), the coefficient of inbreeding was estimated to be ∼1/95. D: Simplified pedigree showing that the parents of the patient are second-cousins. Black shading indicates primary microcephaly and hearing loss. The patient is the fifth child in a sibship of six. We note that the male–male link in this consanguineous loop means that the homozygous region on Xq22.3 is unlikely to have come from these great-great-grandparents. E: Sanger sequencing identified a homozygous chr9:123,292,381C>A mutation, inherited from the heterozygous mother. Electropherogram shows sequence on the negative (i.e., coding) strand so mutation appears as G>T and predicts a Glu → STOP codon. DNA from the father and the patient's siblings was not available.

The patient was born to Somali parents at 40 weeks gestation, weighing 2.37 kg (−2.39 SDS) with an occipito-frontal head circumference (OFC) of 30cm (−3.68 SDS) and length of 43 cm (−3.68 SDS). Auditory brainstem response testing at 3 days of life was normal. Although there were no neonatal problems, she experienced significant gastro-oesophageal reflux during the first 4 months and was fed via a gastrostomy (aged 9 months). Early developmental milestones were delayed. A brain MRI performed at 15 months showed microcephaly but no structural abnormalities (Fig. 1A). Development was assessed aged 3 using the Vineland Adaptive Behaviour scale: function was between the 1st and 6th percentiles for various domains of development with communication, motor, and composite adaptive behavior skills on the 1st centile and social and daily living skills on the 6th and 5th centiles, respectively. Moderate-to-severe bilateral sensorineural hearing loss was diagnosed at 3 years, 10 months. Molecular testing of *GJB2* and *GJB6* was normal. Although language skills progressed following the introduction of hearing aids, at 6 years she communicates largely by gestures. She is described as a happy, sociable child with no reported behavioral problems. At 6 years, growth parameters were: weight 17.6 kg (−1.12 SDS), height 107.5 cm (−1.61 SDS), and OFC 41.5 cm (−8.91 SDS), with a sloping forehead (Fig. 1B). There was mild joint laxity, hypotonia, and decreased muscle bulk. No maternal or environmental causes were identified that may have contributed to the microcephaly or deafness. Given the absence of associated malformations or neurological deficits, a clinical diagnosis of primary microcephaly was made. *ASPM* was sequenced but no mutations were identified.

## MATERIALS AND METHODS

### Genotyping

With appropriate ethical approval and consent, DNA from the patient and mother were genotyped using the CytoSNP-12 v2.1 array (Illumina Inc., San Diego, CA). DNA from the patient's father could not be obtained. Data analysis was performed with Nexus v5.1 Discovery Edition (BioDiscovery, Hawthorne, CA) and GenomeStudio V2009.2 (Illumina).

### Sequencing

All 38 exons and intron–exon boundaries of the *CDK5RAP2* gene were amplified using the FastStart Taq DNA polymerase (Roche, Burgess Hill, UK) and primers from a previous study [Hassan et al., 2007]. PCR products were purified using exonuclease I (NEB, Ipswich, MA) and shrimp alkaline phosphatase (USB, Cleveland, OH). Bidirectional Sanger sequencing was then performed using BigDye chemistry (Applied Biosystems, Foster City, CA) and run on a 3730xl DNA Analyzer (Applied Biosystems).

## RESULTS

The genome-wide SNP analysis did not reveal any copy number variants (CNVs) in the patient, other than those noted already in the Database of Genomic Variants. However, two large tracts of cnLOH involving chr7p15.2–p21.3 and chr9q33.1–q34.12 (Fig. 1C) were noted of greater than 5 Mb. These were not observed in the mother. Co-occurrence of two independent cnLOH regions led us to consider an unreported consanguineous parental relationship. The coefficient of inbreeding (fraction of the genome showing cnLOH) was estimated to be ∼1/95, consistent with parents who are second-cousins or second-cousins-once-removed. Subsequent re-evaluation of family-history established that parents were indeed second-cousins (Fig. 1D).

Of the eight known microcephaly genes, one (*CDK5RAP2*) is situated within the candidate region on chr9q33.1–q34.12. Targeted sequencing of this gene revealed three homozygous coding changes. Two of these were common missense polymorphisms (rs4837768 and rs4836822), whereas the third was a novel nucleotide transversion in exon 8 (c.700G>T, NM_018249.4) that predicts a premature stop at codon 234 (Fig. 1E). The variant was heterozygous in the mother but was not seen in >5,000 Caucasian and African-American samples in the Exome Variant Server (http://evs.gs.washington.edu/EVS/; v.0.0.10).

We next considered the possibility that one of the cnLOH loci might harbor a second rare mutation, in a deafness gene. In addition to the large regions of cnLOH on chr7p and chr9q, five other cnLOH loci of intermediate size (2–5 Mb) were identified. These were located at 3p24.3, 4q26, 5q21.1, 16p11.2 (peri-centromeric region), and Xq22.3. We searched these seven loci for genes noted in OMIM as being linked with deafness and although we identified four genes (*DFNA5*, *HOXA2*, *COL4A5*, and *PRPS1*), none of these appeared to be a likely candidate based on their inheritance pattern or because the phenotype of our patient did not match.

## DISCUSSION

To date, only two disease-causing mutations in *CDK5RAP2* have been described, both in consanguineous families; a Y82X mutation [Bond et al., 2005; Hassan et al., 2007] and a IVS26-15A>G splicing mutation [Bond et al., 2005] (Table I). All three published families are from Northern Pakistan. Therefore, our report of a Somali child with primary microcephaly and a novel E234X mutation confirms *CDK5RAP2* as a disease gene with clinical relevance outside the Pakistani population.

Common features of patients with *CDK5RAP2* mutations include microcephaly from birth (−4 to −8 SDS), with mild-to-moderate learning disability (Table I). All have a short sloping forehead but otherwise there do not appear to be any other dysmorphic features or associated malformations in common. Notably, our patient has significant bilateral sensorineural hearing loss. This manifestation is not generally considered to be consistent with autosomal recessive microcephaly and therefore it is highly possible that the deafness may be caused by a further gene mutation, independent of *CDK5RAP2*. We speculated that such a mutation, in a consanguineous family like this, might be recessive, caused by an additional homozygous mutation. However, when we scanned the seven cnLOH regions for phenotypes entered in OMIM, we identified only one autosomal recessive hearing loss phenotype (OMIM #612290: microtia, hearing impairment, and cleft palate caused by *HOXA2* mutations), which did not match the phenotype of our patient. There were two deafness genes on Xq22.3; *COL4A5* which is implicated in Alport syndrome with variable sensorineural hearing loss [Barker et al., 1990] and *PRPS1* which is mutated in non-syndromic X-linked deafness-1 [Liu et al., 2010]. However, since all five siblings of the proband (two males and three females) and her parents are clinically normal, we believe that both genes are unlikely candidates for the progressive sensorineural hearing loss noted in the proband. We also identified an autosomal dominant non-syndromic sensorineural deafness phenotype (OMIM #600994 caused by *DFNA5* mutations) and we have not ruled out the possibility of a de novo mutation in this or other dominant deafness genes in our patient. Other explanations for the deafness include the existence of a novel recessive deafness gene located in one of the cnLOH regions. Alternatively, there is a possibility of compound heterozygous mutations elsewhere in the genome which would not have been detected by our autozygosity mapping approach. Interestingly, a recent study unexpectedly detected multiple disease alleles at the *DFNB3* locus within a single consanguineous pedigree [Lezirovitz et al., 2008]. The increasing use of exome sequence data now allows filtering for genetic variants that are compatible for multiple disease mechanisms and a recent study used this method to resolve a complex case of Miller syndrome to be a combination of two different genetic disorders [Ng et al., 2010].

The hearing loss present in individual VI:3 published previously [Moynihan et al., 2000] prompted us to also consider the possibility that *CDK5RAP2* mutations might have variable expressivity and that this might extend to include sensorineural hearing loss. However, in the case described previously, the deafness was congenital, whereas our patient passed a hearing test at birth, suggesting different etiologies. Nevertheless, we cannot rule out a link based on these cases alone and therefore further detailed clinical descriptions of rare *CDK5RAP2* patients, including hearing assessments, will be needed to help resolve the full phenotypic range associated with mutations in this gene.

In a recent array-CGH study, 22% of subjects with brain malformations carried rare CNVs, many of which are likely to have etiological relevance [Kariminejad et al., 2011]. In our study, insufficient DNA was available for standard array-CGH. The decision to test the patient using a SNP-array (requiring significantly less DNA) instead proved fortuitous and highlights the advantage of SNP-arrays over array-CGH platforms in helping to guide targeted sequencing efforts, especially in cases where a condition is suspected to be recessive and parental consanguinity is a possibility. Whilst both platforms can detect pathogenic CNVs, only the SNP platform can detect all forms of cnLOH [Bruno et al., 2011], inform subsequent targeted sequencing strategies and if needed, confirm family relationships through SNP genotypes. In this way, SNP-array testing can also act as an invaluable prescreening and supportive tool when embarking on expensive whole genome and exome sequencing studies. However, it should be cautioned that using genomic data to infer family relationships can be a sensitive issue [Schaaf et al., 2011] and appropriate genetic counseling should be provided prior to testing.

In summary, we identified a novel *CDK5RAP2* mutation, the first in a patient of non-Pakistani descent. Our study confirms *CDK5RAP2* is a rare primary microcephaly disease gene and emphasizes that when no consanguinity is reported but is suspected, SNP-array testing can reveal cnLOH that may infer distant relationships between parents and guide disease gene identification.
